# Risk of venous thromboembolic disease and adequacy of prophylaxis in hospitalized patients in Argentina: a multicentric cross-sectional study

**DOI:** 10.1186/1477-9560-12-15

**Published:** 2014-07-07

**Authors:** Fernando Vazquez, Ricardo Watman, Aldo Tabares, Carina Gumpel, Enrique Baldessari, Alicia B Vilaseca, Federico J Capparelli, Esteban Lifschitz

**Affiliations:** 1 Internal Medicine Department, Hospital Italiano de Buenos Aires, Juan D. Perón 4190, (C1181ACH), Buenos Aires, Argentina; 2 Chief of Medical Policies, Swiss Medical Medicina Privada, Buenos Aires, Argentina; 3 Vascular Medicine and Thrombosis Department, Hospital Privado, Córdoba, Argentina; 4 Hematology Department, Sanatorio Plaza, Rosario, Argentina; 5 Internal Medicine Department, Hospital Universitario, Fundación Favaloro, Buenos Aires, Argentina; 6 Hematology Department, Clínica San Camilo, Buenos Aires, Argentina; 7 Internal Medicine Department, FLENI, Instituto de Investigaciones Neurológicas Raúl Carrea, Buenos Aires, Argentina; 8 Internal Medicine, Clínica Santa Isabel, National Coordinator of the Thrombosis-Free Area Argentina Program, Buenos Aires, Argentina

**Keywords:** Thrombosis, Thromboprophylaxis, Venous thromboembolic disease, Adequacy, Pulmonary embolism, Deep vein thrombosis

## Abstract

**Background:**

Venous thromboembolic disease (VTE) is associated with high morbi-mortality. Adherence rate to the recommendations of antithrombotic prophylaxis guidelines (ATPG) is suboptimal. The aim of this study was to describe the adequacy of antithrombotic prophylaxis (ATP) in hospitalized patients as the initial stage of a program designed to improve physician adherence to –ATP recommendations in Argentina.

**Methods:**

This study was a multicenter, cross-sectional study that included 28 Institutions throughout 5 provinces in Argentina.

**Results:**

1315 patients were included, 729 (55.4%) were hospitalized for medical (clinical) reasons, and 586 (44.6%) for surgical reasons. Adequate ATP was provided to 66.9% of the patients and was more frequent in surgical (71%) compared to clinical (63.6%) subjects (p < 0.001). Inadequate ATP resulted from underuse in 76.6% of the patients. Among clinical, 203 (16%) had increased bleeding risk and mechanical ATP was used infrequently.

**Conclusions:**

The adequacy of ATP was better in low VTE risk clinical and surgical patients and high VTE risk in orthopedic patients. There was worse adequacy in high risk patients (with active neoplasm) and in those with pharmacological ATP contraindications, in which the use of mechanical methods was scarce. The adequacy of ATP was greater at institutions with < 150 beds compared with larger institutions. This is the first multicentric study reporting ATP in Argentina. Understanding local characteristics of medical performance within our territory is the first step in order to develop measures for improving ATP in our environment.

## Background

Venous thromboembolic disease (VTE) is associated with high morbidity and mortality. Approximately 2 million cases of deep venous thrombosis (DVT) and 200,000 deaths from pulmonary embolism (PE) are reported annually in the United States [1]. These numbers exceed those from breast cancer and AIDS deaths combined [2-4]. Additionally, VTE affect quality of life during both acute and chronic phase [5,6], due to the disease itself and the bleeding risk as a complication of anticoagulation.

Despite its health impact [1,7] and the availability of evidence-based antithrombotic guidelines (ATPG) on prophylaxis [8-10], the adherence rate to such recommendations has been reported in several studies as being suboptimal [11,12].

Although there are reports on the adequacy of antithrombotic prophylaxis (ATP) in different countries, in Argentina there are only two reports in two local hospitals that describe ATPG adherence [13,14]. This paper describes the adequacy of ATP guideline compliance in hospitalized patients as the initial stage of a program designed to improve physician adherence to VTE prophylaxis recommendations.

### Primary objective

To determine the proportion of patients at risk of VTE who receive appropriate, recommended ATP.

### Secondary objective

To evaluate the association between the different characteristics within participating institutions and adherence to antithrombotic guidelines on prophylaxis.

## Methods

### Design

Descriptive cross-sectional study.

### Setting

The survey was conducted at 28 institutions located in 5 provinces of Argentina. The institutions were classified according to the presence of training medical residents and categorized according to the number of beds (less or more than 150 beds). The proportion of admitted patients with elevated risk (APER) of VTE (> or ≤ 70%) in each Institution was also classified. The present study involves several Institutions with different case mix of patients. Data of previously published studies from Latin america have shown that 60 to 80% of hospitalized patients belong to moderate to high risk of VTE categories [14,15].

Subsequently, the association between these variables and the adequacy of ATP prescription was assessed.

Each institution had one local coordinator who selected a group who in turn conducted the local survey. To ensure homogeneity of the data collection, all of the participating investigators received the same training.

To avoid prescribing behavioral changes for VTE prophylaxis, the attending physicians were blinded to the purpose or timing of the survey.

A unique form was designed and used by all of the participating Institutions. This form allowed the identification of variables that determined the risk group for each patient as well as the type of prophylaxis received. For the elaboration of the form and to determine the adequacy of the prophylaxis according to the risk of VTE, we used as a frame of reference the American College of Chest Physician (ACCP) guide, which was published in 2008 [9].

The survey was performed during a random day in each institution between June 2009 and October 2011, using a cross-sectional model of all hospitalized patients who had met defined inclusion and exclusion criteria.

At the time of the analysis, the type of hospitalization (medical or surgical), the presence or absence of risk factors, the reason for hospitalization or surgery, the presence of increased bleeding risk and the type of prescribed prophylaxis were considered. For clinical patients, the age, reason for admission and presence of VTE risk factors were considered. The following factors were considered in surgical patients: age, surgery type (programmed vs. emergency), duration, and whether it was related to a neoplasm or not.

Definitions: for the definition of adequate prophylaxis all Institutions created a protocol to guide the prescription of VTE prophylaxis, based on the ACCP guidelines [9,16]. Adequate prophylaxis was considered when 1) the patient had increased VTE risk, low bleeding risk and received pharmacological thromboprophylaxis in adequate doses; 2) the patient had low VTE risk, no indication for ATP, and did not receive prophylaxis and 3) patients had increased VTE and bleeding risk did not receive any pharmacological thromboprophylaxis but received a mechanical one. Within the group of patients with increased VTE risk, there was another subgroup considered as very high risk: patients with active cancer, history of VTE or major orthopedic surgery.

In patients who received inadequate prophylaxis, we differentiate two groups: excessive *inadequate prophylaxis*, when the patient received pharmacological ATP without clinical indication, or when the administered dose was higher than recommended. On the other side, insufficient *inadequate prophylaxis* when the patient had an increased VTE risk but received no prescription for prophylaxis or the prescribed dose was less than recommended [9,16].

Contraindications for pharmacological prophylaxis: platelets count < 50.000/ml, active bleeding, recent (in the last 7 days) major bleeding, severe renal failure (< 30 ml/min creatinine clearance), coagulopathy (including disseminated intravascular coagulation, coagulopathy related with sepsis, liver failure, hemophilia, von Willebrand disease, any severe laboratory alteration in coagulation test), active peptic ulcer, contraindication related with invasive procedures.

The data was obtained from medical records and patient prescriptions without direct contact between the investigator and the patient or the treating physician.

### Inclusion criteria

Hospitalized patients aged 21 years or older.

### Exclusion criteria

Pregnancy, postpartum within the last month, patients participating in another clinical study, patients receiving anticoagulants for any reason, patients hospitalized because of DVT or PE and those hospitalized in intensive care units.

Each form was recorded in Access® for future analysis. The study was performed in compliance with the Helsinki Declaration and was approved by the Ethics Committee of each Institution. An oral informed consent was obtained from each patient, as required in Argentina for observational designs.

### Statistical analysis

All of the data analyses were performed with IBM SPSS software, version 19 (SPSS, Chicago, Illinois). Continuous variables are described as mean with their standard deviations, and categorical variables are described as proportions with 95% CI. The means were compared with a *t*-test, and the proportions were compared with χ^2^ or Fisher’s exact tests. A statistical significance was considered when p < 0.05. To assess factors associated with adequate prophylaxis a multiple logistic regression was used. Variables that were statistically significant in univariate analysis and those not significant but with clinical relevance were included in it. Results are expressed as OR and 95% CI for each explanatory variable. The goodness of fit of the model was assessed with Hosmer-Lemeshow.

## Results

**Figure 1:**
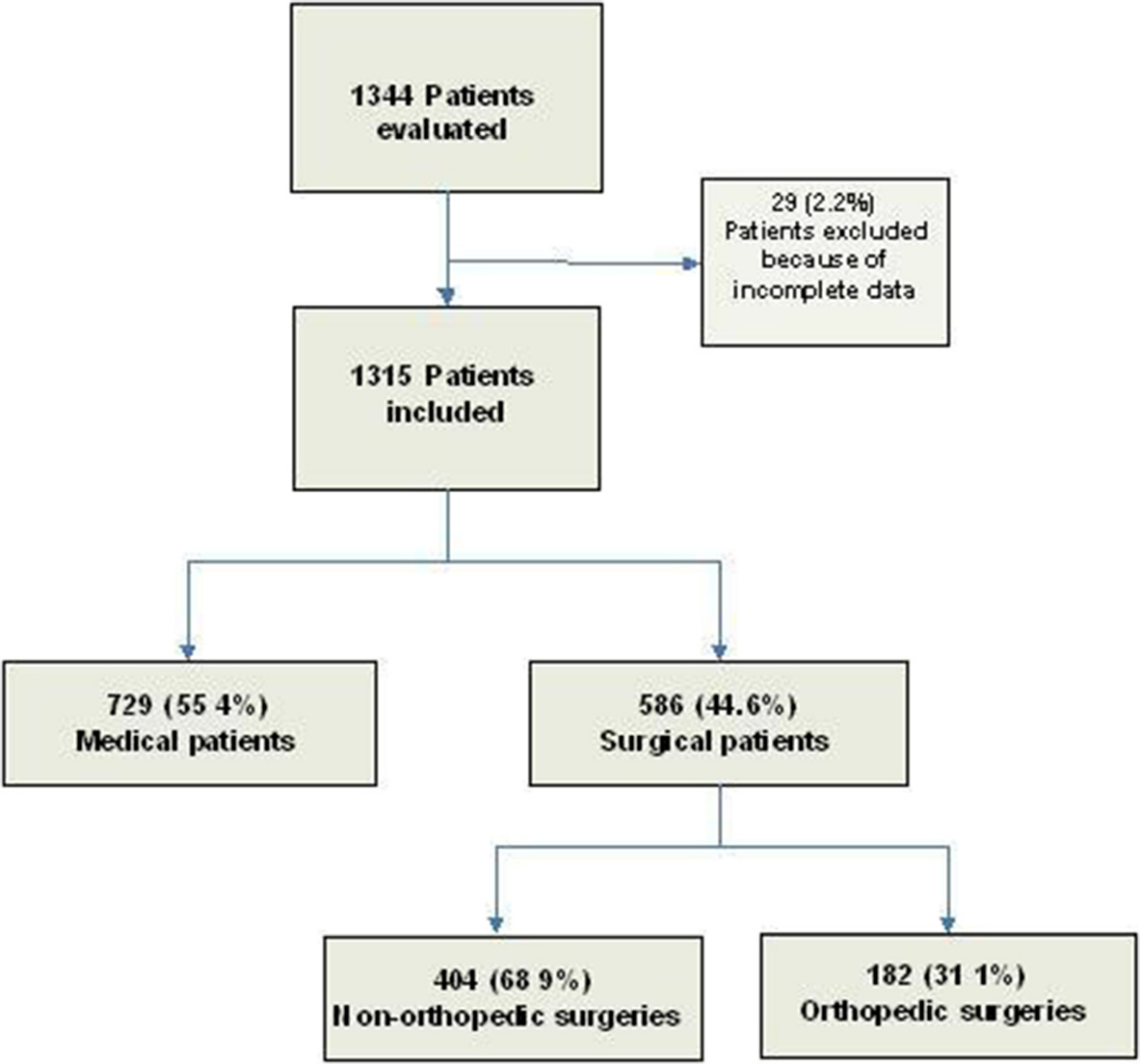
Flowchart of patients evaluated in the study.

Of 1344 patients recorded, 29 (2.2%) were excluded because of missing data, and 1315 were included in the analysis. There were 729 (55.4%, CI 95% 53–58) patients hospitalized for clinical reasons and 586 (44.6%, CI 95% 42–47) hospitalized for surgical reasons, of whom, 182 (31.1%) were orthopedic surgeries and 404 (68.9%) were non orthopedic (Figure 1).

Mean age was 61.5 (SD 18.7) years, and 677 (51.5%, CI 95% 49–54) patients were male. Baseline characteristics and their primary risk factors (RFs) for VTE are shown in Table 1.

**Table 1:** Baseline characteristics of the population

**Population**	**Total n = 1315**	**Clinical n = 729**	**Surgical n = 586**	**p -value**
Age (years)*	61.5 (18.7%)	64.5 (18.7%)	57.8 (17.9%)	< 0.001
Male gender N (%)**	677 (51.5%, 49-54)	384 (52.7%) 49-56)	293 (50.0%) (46-54)	NS
**Risk factors n (%)**				
Prolonged rest or lower limb paralysis	483 (36.7%) (34-39)	330 (45.3%) 42-49	153 (26.1%) (23-30)	< 0.001
Active cancer and/or chemotherapy treatment	272 (20.7%) (18-23)	159 (21.8%) (19-25)	113 (19.3%) 16-22	NS
Overweight (BMI > 25)	267 (20.3%) (18-22)	330 (45.3%) (17-23)	123 (21%) (18-24)	NS
Varicose veins or chronic venous insufficiency	112 (8.5%) (7-.1)	68 (9.3%) (7-11)	44 (7.5%) (5-10)	NS
Heart failure	111 (8.4%) (7-10)	85 (11.7%) (9-14)	26 (4.4%) (3-6)	< 0.001
Central venous catheter	116 (8.4%) (7-10)	61 (8.4%) (63-10)	55 (9.4%) (7-12)	NS
Chronic obstructive pulmonary disease	78 (5.9%) (5-7)	61 (8.4%) (6-10)	17 (2.9%) (2-4)	<0.001
History of DVT/PE	27 (2.1%) (1-3)	21 (2.9%) (2-4)	6 (1.0%) (2-2)	0.018
Myeloproliferative syndrome	18 (1.4%) 7-2)	17 (2.3%) (1-3)	1 (0.2%) (2-5)	< 0.001
Inflammatory disease	15 (1.1%) (0.57-1.7)	11 (1.5%) (1-2)	4 (0.7%) (0.1-1.3)	NS
Estrogen therapy	14 (1.1%) 0.5-1.6	7 (1%) (0.2-2)	7 (1.2%) (0.32)	NS
Nephrotic syndrome	12 (0.9%) (0.4-1.4)	10 (1.4%) (1-2)	2 (0.3%) (0.1-0.8)	NS
Thrombophilia	5 (0.4%) 0.05-0.7	3 (0.4%) (0.1-0.9)	2 (0.3%) (0.13-0.8)	NS

*Expressed as the mean and standard deviation (SD).

**Expressed as proportions and the confidence interval.

Five hundred eleven (38.9%, CI 95% 36–41) patients had one RF for VTE, 269 (20.5%, CI 95% 18–23) had 2 RFs, 149 (11.3%, CI 95% 9.6-1.3) had 3 or more RFs and no RFs were found in 386 (29.4%, CI 95% 9.6-1.3) patients.

A head-to-head comparison of the clinical and surgical patients showed that clinical patients were older, exhibited more prolonged immobility and/or paralysis of one or more extremities and suffered from congestive heart failure, chronic obstructive pulmonary disease and myeloproliferative syndromes. The coexistence of 2 or more RFs for VTE was more common in the clinical than in the surgical group (78% vs. 61%). The presence of an active neoplasm was common in both groups; with a prevalence of 21.8% (CI 95% 19–25) in clinical and 19.3% (CI 95% 16–22) in surgical patients (Table 1).

Considering the entire analyzed population, 880 (66.9%, CI 95% 64–69) patients received adequate ATP. However, ATP was more appropriately indicated in surgical patients, 416 (71%, CI 95% 67–75) than in clinical patients, 464 (63.6%, CI 95% 60–67), (p <0.001).

Among the 435 (33.1%, CI 95% 31–36) cases of inadequate ATP, both in the global cohort and in each separate group (clinical and surgical), ATP was insufficient in 76.6% (CI 95%.73-81) and excessive in 23.4% (CI 95% 20–27).

Low-molecular weight heparins (LMWH) were the most frequent pharmacological ATP drug used, in 569/873 (65.1%, CI 95% 62–68) of the total cases, similar among clinical and surgical patients.

Unfractionated heparin (UFH) was the second most commonly used ATP strategy in 163/873 (18.7%, CI 95% 16–20) patients. UFH was not more significantly used in clinical 107 (14.7%, CI 95% 12–17) than in surgical patients 56 (9.6%, CI 95% 7–12). New oral anticoagulants (NOA) were prescribed less commonly.

One or more bleeding risk factors were identified in 213 (16.2%) cases, and this issue occurred more commonly in clinical patients. Bleeding risk factors included severe renal failure in 71 (33.3%) patients, active bleeding in 67 (31.5%) patients and thrombocytopenia in 57 (26.8%) patients. Thrombocytopenia was the only factor more commonly found in clinical patients (30.6% vs. 11.6%, p = 0.015).

Infrequent bleeding risk factors were coagulation disorders (9.9%) central nervous system hemorrhage (7%) and hepatic insufficiency (5.2%). Of the 213 patients with bleeding risk factors, ATP was adequate in 107 (50.2%). One hundred and sixty patients showed absolute contraindications for pharmacological ATP; and only 23 (13.8%) received adequate mechanical ATP. A hundred and thirty cases had a high risk for VTE in this group but the ATP prescribed was adequate in only 42 (32.3%) patients.

Mechanical ATP measures were rarely used. Graded compression stockings (GCS), were indicated in 70 (5.3%, CI 95% 4–7) patients, and intermittent pneumatic compression in 13 (1%, CI 95% 0.5-2). GCS were used more commonly in the clinical group (7.1% vs. 2.4%, p < 0.001).

Early ambulation was indicated in 229 (17.5%, CI 95% 15–19) cases and was significantly more common in the surgical (25.6%, CI 95% 22–29) than in the clinical group (11%, CI 95% 9–13, p < 0.001).

### Clinical patients

Of the 729 patients with clinical conditions, 430 (59%, CI 95% 55–63) exhibited total immobility or had only bathroom privileges while hospitalized and were associated with at least one other RF for VTE. The three leading causes of hospitalization were acute infection (39.3%, CI 95% 35–44), cancer complications (30.7%, CI 95% 26–35) and stroke (22.3%, CI 95% 18–26).

Among the patients hospitalized for clinical reasons, 620 (85%) should have received ATP. However, ATP was adequate in only 464 patients (63.6%, CI 95% 60–67) and inadequate in 265 (36.4%, CI 95% 33–40). In 203 cases (76.6%, CI 95% 72–82) the main reason for inadequacy was underuse and in 62 (23.46%, CI 95% 18–28) was excessive use. In the clinical group 129 (20.8%) had contraindications for receiving pharmacological ATP. No need to receive ATP was present in 109 cases (15%) of low VTE risk; this group was adequately treated without ATP in 79 cases (72.5%), but ATP in excess was prescribed in 30 cases (17.5%).

Among the patients meeting criteria for receiving ATP, 385/620 (62.1%) cases received adequate ATP and, among those without any criteria for receiving ATP (low risk of VTE), ATP was prescribed adequately in 79/105 (72.5%) cases. The difference between the two groups was statistically significant (p < 0.001).

### Surgical patients

**Figure 2:**
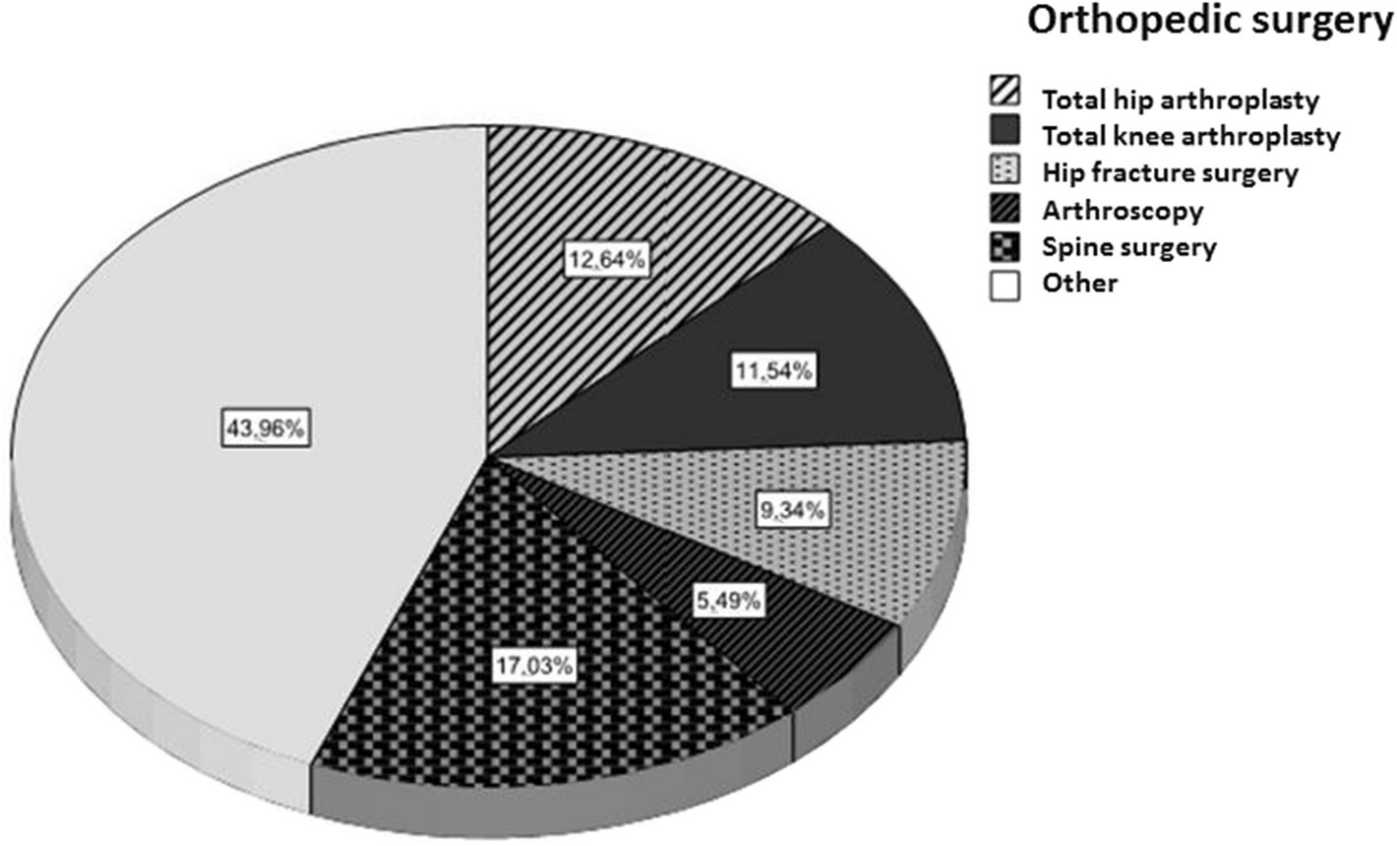
Types of orthopedic surgical procedures.

**Figure 3:**
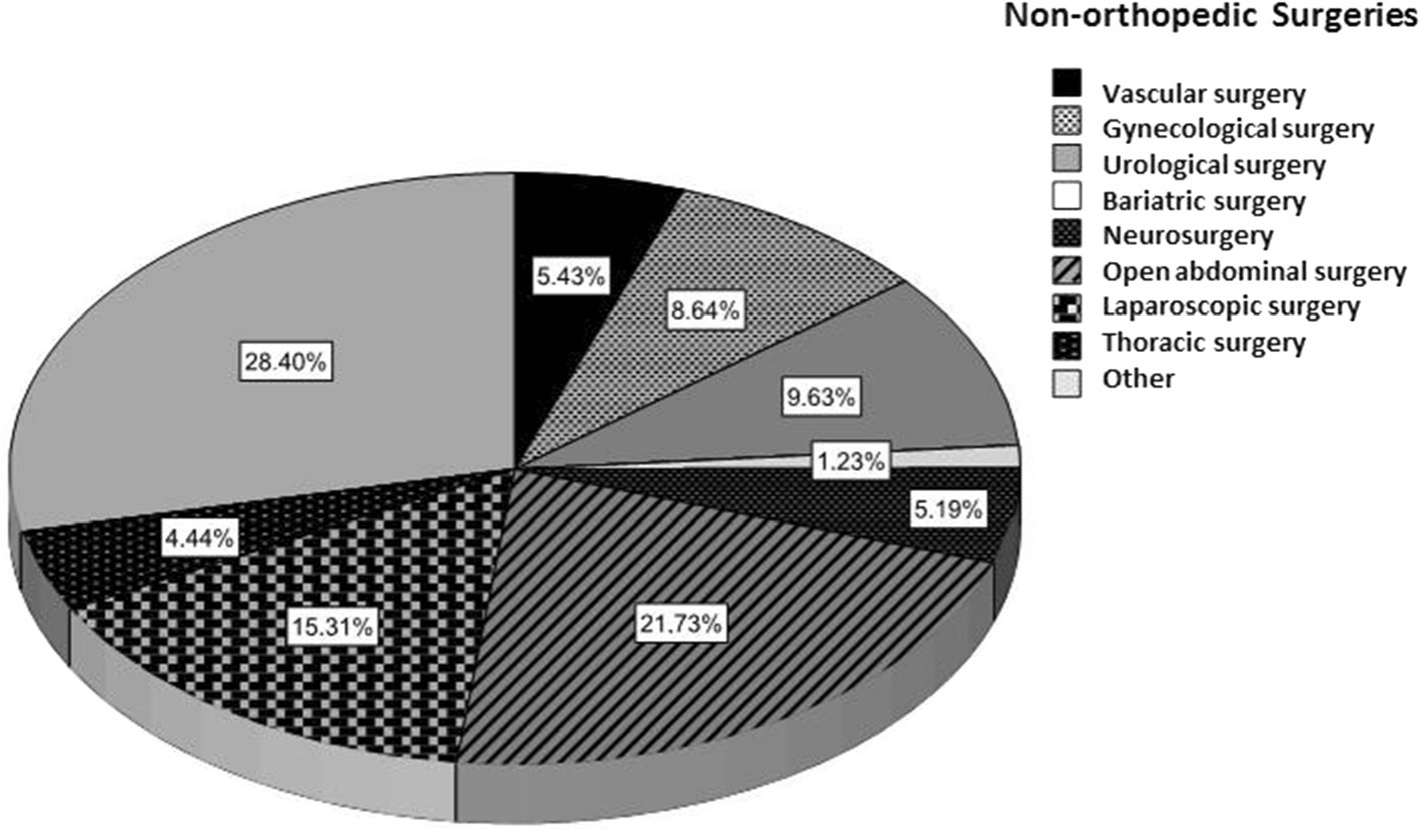
Non-orthopedic surgical procedures.

Of the 586 patients with surgical conditions, 384 (65.4%, CI 95% 62–69) had undergone an elective surgery, 420 (71.6%, CI 95% 68–75) had undergone procedures lasting more than 45 minutes, 31% had undergone orthopedic surgery, and surgery was indicated in 95 (16%, CI 95% 13–19) patients because of oncologic conditions. The types of surgical procedures are shown in Figures 2 and 3. The different types of surgeries surveyed are outlined in Figures 2 and 3.

### Subgroup of orthopedic surgery

The 182 patients with orthopedic surgical conditions were classified into high or low risk for thrombosis; in the high risk group (hip or knee prosthesis and hip fracture) 57/61 (93.4%, CI 95% 87–99) received adequate ATP. In the remaining 121 with low to moderate VTE risk prophylaxis was adequate in 90 (74.4%, CI 95% 67–82) cases.

### Subgroup of general surgery

In this group of 404 patients, 238 (59%, CI 95% 50–59) had high-risk of VTE, 39 (9.6%, CI 95% 7–13) had moderate-risk and 127 (31.4%, CI 95% 31–40) had low-risk. Overall, 267 (66.1%) patients received adequate ATP. In 120 cases, (83.6%) ATP was insufficient and this situation was significantly more common in the VTE high risk group, 103 (43.3% CI 95% 37–50) and 16 with moderate risk (40.9%, CI 95% 26–55) patients (Table 2).

**Table 2:** Prophylaxis in non-orthopedic patients according to the risk group

**Risk group**	**Patients (N = 404) 100%**	**Adequate (N = 267) 66.1%**	**Inadequate excessive (N = 17) 4%**	**Inadequate insufficient (N = 120) 29.7%**
High	238 (59%) (50-59)	129 (54.2%) (52-65)	6 (2.5%) (0.5-5)	103 (43.3)
Moderate	39 (9.6%) (7-13)	23 (59.1%) (4.5-7.4)	0 (0)	16 (40.9)
Low	127 (31.4%) (31-40)	115 (90.5%) (83-93)	11 (8.6%) (6-16)	1 (0.8)

p < 0.001.

### Characteristics of the participating institutions

There was no association between the presence of a residency program (p 0.27), nor the percentage of patients at high VTE risk (p 0.057) with the appropriateness of the indications of prophylaxis; however, institutions with fewer beds (p 0.027) had higher frequency of appropriate prophylaxis.

Table 3 shows the variables assessed in the multiple logistic regression model. Institutions with more than 150 beds, patients with active cancer and those with contraindicated anticoagulation were associated with inadequate ATP, while major orthopedic surgery was associated with better adequacy of ATP.

**Table 3:** Variables evaluated with multiple logistic regression model for appropriate prophylaxis

**Variable**	**OR**	**CI 95%**
Age	0.99	0.99-1.01
Length of stay	1.01	0.783-1.31
History of VTD	1.25	0.52-3.03
Active cancer	0.63	0.47-0.83
Major orthopedic surgery	5.80	2.05-16.43
APER (>70%)	1.15	0.89-1.49
Number of beds (>150)	0.74	0.57-0.95
Residency	1.20	0.88-1.63
Contraindication for pharmacological prophylaxis	0.34	0.24-0.47

OR: odds ratio; CI 95%: confidence interval; VTD: venous thromboembolic disease; APER: proportion of Admitted Patients with Elevated Risk > 70%.

## Discussion

This study shows that approximately 70% of the patients admitted to general hospitals in different provinces in Argentina presented risk factors for VTE and, therefore, should have received antithrombotic prophylaxis.

One-third of the patients showed several combined risk factors for VTE; prolonged bed rest, cancer and obesity were the three most commonly observed, consistent with the report of Cohen et al. [11].

The primary causes for hospitalization due to medical conditions included respiratory conditions, infections, malignancy and acute neurological conditions, consistent with previous reports [14]. The most frequent surgeries were orthopedic and both conventional and laparoscopic abdominal surgery, followed by urological surgery.

Similar to what Gladding et al. [17] found, the proportion of patients with medical conditions and with increased risk for VTE, was greater than the proportion of surgical patients with increased risk (84.2% vs. 67.6%) (p < 0.001). Adequacy of ATP was higher in the low risk medical group of patients.

The use of ATP was adequate in 66.9% of the total patients surveyed and was more common among the patients with surgical conditions (particularly in high-risk orthopedic surgeries) than among those hospitalized with medical conditions. If non-surgical patients are taken into account, the adequacy of ATP was higher than previously reported [18,19].

In patients undergoing orthopedic surgery, ATP was more appropriate in patients at high risk for VTE, unlike general surgical patients, in whom the adequacy of ATP was higher in patients categorized as low risk.

The better compliance in the surgical group in comparison with those in the medical group may be related to the fact that it is simpler to categorize the risk for VTE in patients with surgical conditions (particularly in major orthopedic surgeries) than in those with clinical conditions and that the benefits of prophylaxis in patients with clinical conditions have been shown more recently than the benefits for surgical conditions.

In this study the adequate thromboprophylaxis was higher than the average reported in the literature [11,12].

It is important to emphasize that the result of the inadequacy in the administration of pharmacological prophylaxis has two consequences: in the case of underutilization, the risk of VTE is increased; and when used in excess, can increase the risk of bleeding. Regarding the two previous studies performed in Argentina [13,14], adherence rates of ATP in this study were lower. Previous studies were conducted in a single center, with fewer beds, facilitating a tighter control of the prescriptions. Moreover, in one study, physicians were not unblinded to the survey, and this could be interpreted as a bias, to proper indication for thromboprophylaxis (13).

At least one factor that increased bleeding risk was observed in 16.2% of the patients surveyed which was greater than reported by Vallano et al. [19]; most of these patients had been hospitalized for clinical conditions. One-third had an increased bleeding risk because of severe renal failure with a creatinine clearance < 30 mL/min. The remaining patients had formal contraindications for receiving pharmacological antithrombotic prophylaxis. The number of patients with contraindications for ATP was similar to previous reports [11,12,14].

There are two situations in which there is a troubling lack of adherence to ATP.

First, the antithrombotic prophylaxis was adequate in only half of the patients with an increased risk of VTE and absolute contraindication for pharmacological ATP. In this regard, it is interesting to note the scarce use of mechanical prophylaxis in this group of patients. Secondly, in surgical patients being operated for cancer (16.5%), who were at very high thrombotic risk only 55.6% of these patients received adequate ATP. These two situations are probably the ones generating more concern and efforts should be targeted to improve safety in ATP in these subgroups.

The analysis of the causes for inadequate ATP revealed that it was primarily caused by overuse in patients within the low-risk category, while underuse was observed in both high-risk and moderate-risk patients. Across all institutions, the most common type of pharmacological ATP used was LMWH followed by UFH.

Of note is that smaller institutions prescribe ATP more adequately reflecting the greater feasibility of appropriate control of thromboprophylaxis prescriptions and with greater feedback when inadequate ATP prescription is made.

VTE has been identified as the number one cause of preventable death among hospitalized patients, and one of the most important causes is contrast between our in-depth knowledge of how to prevent VTE and our lackadaisical implementation of prophylactic measures, which is unacceptable. In 2008, Medicare declared the occurrence of VTE after total knee or hip replacement to be a “never event.” This means that hospitals will have to pay for the extra costs of treating postoperative DVT or PE following knee or hip replacement [20].

Regarding compliance with the indications for ATP according to the recommendations in different patient groups, we believe that local awareness in our country is of utmost importance when interventional measures begin; the objective should be to improve patient risk stratification, thereby optimizing the appropriate indication for ATP.

Once we identified thromboprophylaxis to be suboptimal, we consider it necessary to implement multiple strategies (continuing medical education that involve all health personnel, monitoring and feedback, electronic support systems for decision making [21-23], etc.) to improve adherence to the prophylaxis guidelines in Argentina, with the objective of enhancing the safety of hospitalized patients, especially in the high risk group.

Limitations: Because this is a cross-sectional study conducted at different times in various institutions, some may have had more time than others to become familiar with the guidelines and recommendations that could influence the appropriateness of their prescriptions. This study also has strengths: it was performed by similarly trained physicians to collect data, and these were analyzed by the same person (EL) to achieve uniformity in the processing and interpretation between the different institutions. The study demonstrates the everyday practice in a large sample of the Argentine health system. Considering that no action has been taken yet for improvement, values of adequacy of prophylaxis are above the average of other countries in Latin America.

Conclusion: This is the first multicenter cross-sectional study performed in different provinces of Argentina to asses ATP guideline compliance. Although this study shows that adequate thromboprophylaxis was higher than in the average reported in the literature, we believe that implement multiple strategies to improve adherence to prophylaxis guidelines is necessary in Argentina, with the objective of enhancing the safety of hospitalized patients. The two groups with worse adequacy were those with contraindication for pharmacological prophylaxis, in which no mechanical measures were used and patients undergoing surgery for cancer. In this high risk groups we need to focus especial attention to improve thromboprophylaxis.

## Abbreviations

VTE: Venous thromboembolic disease; DVT: Deep venous thrombosis; PE: Pulmonary embolism; ATPG: Adherence rate to the recommendations of antithrombotic prophylaxis guidelines; ATP: Adequacy of antithrombotic prophylaxis; APER: Admitted patients with elevated risk; ACCP: American College of Chest Physician; LMWH: Low-molecular weight heparins; UFH: Unfractionated heparin; NOA: New oral anticoagulants; GCS: Graded compression stockings.

## Competing interests

This study received an unrestricted grant from Sanofi S.A.

## Authors’ contributions

Conception and organization: FV, RW, EL, CG, EB, AV, FC,Coordination and patient data: FV, RW, EL, Analysis: FV, EL, RW, Manuscript editing: FV. All authors read and approved the final manuscript.
